# Motor Extinction in Distinct Reference Frames: A Double Dissociation

**DOI:** 10.3233/BEN-2012-110254

**Published:** 2013

**Authors:** Jennifer Heidler-Gary, Mikolaj Pawlak, Edward H. Herskovits, Melissa Newhart, Cameron Davis, Lydia A. Trupe, Argye E. Hillis

**Affiliations:** ^1^Departments of NeurologyJohns Hopkins UniversitySchool of MedicineBaltimoreMDUSA; ^2^Department of RadiologyUniversity of PennsylvaniaPhiladelphiaPAUSA; ^3^Department of Neurology and Cerebrovascular DisordersPoznan University of Medical SciencesPoznanPoland; ^4^Physical Medicine and RehabilitationJohns Hopkins UniversitySchool of MedicineBaltimoreMDUSA; ^5^Department of Cognitive ScienceJohns Hopkins UniversityBaltimoreMDUSA

**Keywords:** Extinction, left body, left space, bimanual motor

## Abstract

*Objective:* Test the hypothesis that right hemisphere stroke can cause extinction of left hand movements or movements of either hand held in left space, when both are used simultaneously, possibly depending on lesion site.

*Methods:* 93 non-hemiplegic patients with acute right hemisphere stroke were tested for motor extinction by pressing a counter rapidly for one minute with the right hand, left hand, or both simultaneously with their hands held at their sides, or crossed over midline.

*Results: *We identified two distinct types of motor extinction in separate patients; 20 patients extinguished left hand movements held in left or right space (left canonical body extinction); the most significantly associated voxel cluster of ischemic tissue was in the right temporal white matter. Seven patients extinguished either hand held in left space (left space extinction), and the most significantly associated voxel cluster of ischemic tissue was in right parietal white matter.

*Conclusions:* There was a double dissociation between left canonical body extinction and left space motor extinction. Left canonical body extinction seems to be associated with more dorsal (parietal) ischemia, and left canonical body extinction seems to be associated with more ventral (temporal) ischemia.

## 1. Introduction

Left-sided extinction with double simultaneous stimulation occurs when a patient can detect a stimulus on the side contralateral to the lesioned hemisphere, but fails to detect the same stimulus when presented simultaneously on the side ipsilateral to the lesion. Extinction has generally been considered a mild form of neglect [37], although a double dissociation between visual neglect and visual extinction has been reported [54]. Extinction can be observed in visual, tactile, or motor modalities after right hemisphere stroke [2]. These distinct modalities of extinction have different neuroanatomical correlates [14]. In a previous paper, we reported the surprising finding that left motor extinction (failure to move the left hand or early discontinuation of left hand movements only when the right hand was moving simultaneously) in patients with acute right hemisphere stroke was associated with infarct and/or hypoperfusion (tissue dysfunction) in right superior temporal cortex [14]. Although this area has been reported as frequently associated with hemispatial neglect [20,22], or stimulus/object-centered hemispatial neglect but not viewer-centered neglect [16], it was surprising that superior temporal cortex ischemia was associated with *motor* extinction rather than visual or tactile extinction. We speculated that motor extinction reflected extinction of the canonical left side of the body (a specific type of object-centered or allocentric neglect), irrespective of where the hand was placed in space relative to the midline of the viewer (or person) making the movements.

ISSN 0953-4180/13/$27.50 © 2013 – IOS Press and the authors. All rights reserved

We selected tests of motor extinction to disambiguate the left side of the body (e.g., left hand in either side of space defined by the patient's midline) from the left side of space (e.g., either hand held in left side of space) previously [14], and also in the current study. However, originally we were not able to adequately analyze corresponding lesion sites in the two types of motor extinction (left canonical body and left-space motor extinction) because only one patient (of 88) displayed left space motor extinction. Yet both types of motor extinction may occur, and might be associated with distinct lesions and/or functional networks. Although there have been occasional investigations of extinction in different reference frames [35], and several investigations of neglect in different reference frames (e.g. [13,32] investigations of *motor* extinction are uncommon [52]. We are not aware of studies in which investigators have determined whether the patient extinguishes the left side of the body or the left side of space during bimanual motor tasks. One reason for the paucity of in-depth studies of motor extinction is that motor extinction tends to be transient after brain lesions [15], so it is best studied acutely. Another advantage of studying the phenomenon in acute stroke is that it allows us to identify the lesions responsible for the deficit before recovery or reorganization of structure-function relationships. The lesions associated with isolated extinction are often small [15], and other areas of the brain can assume the function of the damaged areas after the acute stage. Identifying the lesions initially responsible for the deficit requires capturing the deficit acutely, and imaging the patient at the same time.

We hypothesized that, depending on the lesion site, right-hemisphere ischemic stroke can cause extinction of either left hand movements or movements of either hand held in left space when both hands are moving simultaneously. We further hypothesized that temporal lesions would cause only left canonical body extinction, whereas more dorsal lesions (frontal or parietal) would cause left space motor extinction.

## 2. Methods

### 2.1. Participants

A consecutive series of 140 consenting patients with acute right supratentoral hemisphere ischemic stroke admitted to a stroke unit at Johns Hopkins Hospital were tested for motor extinction within 48 hours of onset of symptoms. Patients were also administered a battery of hemispatial neglect tests, such as copying a scene, line cancellation, word and sentence reading, and line bisection, described elsewhere [31]. However, the current study focused on performance on the motor clicking task, designed to distinguish canonical left body motor extinction from left space motor extinction [15]. Additional inclusion criteria included: premorbid proficiency in English, right-handedness, and ability to understand task directions. Exclusion criteria included: reduced level of consciousness or ongoing sedation; known uncorrected hearing loss or visual impairment; history of dementia; previous symptomatic stroke or other neurological disease; hemorrhage or significant edema on initial scans; hemiparesis or hemiplegia identified by the neurology team caring for the patient. Of the original 140 patients, 14 patients were excluded for hemiparesis (that had not been detected by the neurology team caring for the patient), defined as: left hand clicking rate $< 50\%$ that of the right hand when both are clicking alone. Sixteen patients were excluded because they did not complete one of the motor extinction trials, often due to fatigue. We attempted to complete all testing within 24 hours of stroke onset; however, we tested some patients between 24 and 48 hours of stroke onset (usually because they were admitted $>$ 24 hours after initial symptoms). Two patients were excluded because MRI was acquired $>$ 24 hours after admission, and 15 patients were excluded due to technical difficulty analyzing the MRI (e.g., motion artifact).

Thus, the final study population included 93 patients, 13.9% with lacunar infarcts, 43.0% with purely subcortical, non-lacunar infarcts, 22.6% with predominantly cortical infarcts, and 20.4% with mixed cortical-subcortical infarcts.

We also studied 25 neurologically normal controls who were comparable in age and gender to the patients (Table 1).

### 2.2. Imaging

Patients underwent MR examination, including diffusion-weighted imaging (DWI), T2 (to provide anatomical landmarks because of higher spatial resolution and to exclude edema), gradient-echo T2*-weighted (to exclude hemorrhage), and fluid-attenuated inversion recovery (FLAIR; to exclude old strokes) within 24 hours of testing. Areas of ischemia were manually drawn on DWI scans and then registered to the MNI atlas by a post-doctoral fellow, blinded to the behavioral data, and these areas were analyzed for association with the two types of extinction to provide a voxel-based analysis of the associated areas.

Table 1
Sex distribution, mean age and clicking rates in patients in each group

| Group | Age (mean± SD) | Gender % female | Volume of Infarct (mean± SD) |
|---|---|---|---|
| Left Body Motor Extinction | 61.0 ± 10.6 | 35% | 11.2cc ± 3.5cc |
| Left Spatial Motor Extinction | 66.6 ± 14.7 | 29% | 8.6cc ± 8.0cc |
| No Extinction | 61.2 ± 15.7 | 50% | 14.5cc ± 2.6cc |
| Normal Controls | 57.7 ± 17.2 | 40% | NA |
| Significant difference | $p = 0.555$ | $X^2 = 2.5$ $P = 0.48$ | $p = 0.11$ |

### 2.3. *Tests of motor extinction*

Consenting patients and controls were asked to press a hand-held counter as quickly as possible for one minute with the right hand alone, left hand alone, or the two hands simultaneously, creating four conditions: (1) right hand clicking alone; (2) left hand clicking alone; (3) right and left hand clicking simultaneously, hands held at sides (uncrossed condition), and (4) right and left hand clicking simultaneously, hands crossed over midline (crossed condition). Hands were held under a bedside table while the movements were performed (so that the patients could not see their hands), and conditions were presented in random order across patients.

We established two criteria for the identification of left canonical body and space extinction (baseline unit of measurement: number of clicks in 60 seconds during unilateral clicking). These criteria were selected to indicate marked difference (50% fewer clicks) with the left (hand or side) in the bi-manual condition compared to the left in the uni-manual condition. Importantly, no control met the criteria for extinction. *Left canonical body motor extinction* was identified when the left contralesional hand clicking rate was reduced by $\geqslant$ 50% (relative to the right) in the uncrossed condition AND crossed conditions. In the presence of left canonical body extinction, the right contralesional hand clicking rate should stay relatively constant, while the left hand rate (when clicking simultaneously with the right hand) should drop off in any space in which it is held. *Left space motor extinction* was identified when the left hand clicking rate (in left space) was reduced by $\geqslant$ 50% relative to the right hand clicking rate in the uncrossed condition AND the right hand clicking rate (in left space) was reduced by $\geqslant$ 50% relative to the left hand clicking rate when clicking in the crossed condition. Thus, in left space motor extinction any hand movements should be extinguished when held in left space during simultaneous clicking. The reduction of 50% was selected because it is a convincing reduction, and no individual of 25 age-matched control subjects met this criterion for extinction. Because each type of extinction required a relative reduction in clicking rate of one hand compared to the other in two conditions, we could not select a single threshold based on mean performance of the normal controls. Rather, this simple cut-off, which separated normal controls from patients with extinction may have missed some patients with subtle extinction, but was unlikely to have identified patients with false positive extinction.

### 2.4. *Statistical analysis*

Rates of right versus left hand movements, alone and simultaneously, were compared across patients with both types of motor extinction and normal controls, using paired t-tests. We also used paired t-tests to compare right and left hand clicking performance in crossed and uncrossed conditions. We used ANOVA to compare age across groups, and Pearson correlations to identify correlations between rates of clicking between the two hands in each condition for each group.

Identification of neural correlates for each type of extinction required a voxelwise analysis of areas of dense ischemia or infarct associated with each type of extinction. lschemic brain tissue was identified visually using high signal on DWI. Infarcts were manually delineated; DWI images and lesions were then registered to a three dimensional brain atlas using an affine algorithm with mutual-information cost function [FSL-FLIRT software [18]]. Once the regions of interest were in standard stereotactic space we computed voxelwise Fisher exact statistics to determine associations between voxel status (normal/lesion) and motor extinction (normal/deficit) for each voxel for the entire brain.

## 3. Results

There were no significant differences by one-way ANOVA between patients with either type of motor extinction, and those with no motor extinction, or 25 neurologically healthy controls in age or lesion size (Ta-

Table 2
Clicking rates for participants in each group in each condition

| | Left hand clicking rate (#clicks/60s) | | | Right hand clicking rate (#clicks/60s) | | |
|---|---|---|---|---|---|---|
| | Alone | Simultaneously | | Alone | Simultaneously | |
| | | Uncrossed | Crossed | | Uncrossed | Crossed |
| Left body motor extinction | 152 ± 50 | 98* ± 57 | 91* ± 64 | 172 ± 64 | 179 ± 52 | 170 ± 58 |
| Left spatial motor extinction | 159 ± 41 | 121* ± 56 | 155 ± 35 | 186 ± 60 | 179 ± 56 | 157* ± 58 |
| No extinction | 146 ± 46 | 136 ± 54 | 135 ± 54 | 176 ± 48 | 157 ± 43 | 158 ± 49 |
| Normal controls | 148 ± 44 | 139 ± 45 | 142 ± 40 | 180 ± 42 | 156 ± 40 | 162 ± 42 |

*clicking rates that are significantly slower than clicking rate with the same hand used alone within group by paired t-tests, using an alpha level of $p < 0.05$.

ble 1). There was also no significant difference between groups in gender between any two groups by chi square test (Table 1).

Clicking rates in each hand for patients with left canonical body motor extinction, left spatial motor extinction, no motor extinction, and normal controls are shown in Table 2. There were significant differences between groups in the clicking rates only for the left hand used simultaneously with the right in the uncrossed [F(3,114) = 3.0, $p = 0.03$)] or crossed conditions [F (3,114) = 4.86, $p = 0.003$)].

### 3.1. Behavioral results

#### 3.1.1. Left canonical body motor extinction

Twenty of 93 patients met criteria (see the Methods section) for left canonical body motor extinction, by extinguishing left hand clicking when held in left or right space when clicking with both hands. For these 20 patients, there was no significant difference in clicking rates for the left and right hands used independently, although there was a trend for the left hand to be slower ($t = -2.1$; $p = 0.053$), as there was for the controls (Table 2). The left hand was significantly slower when used with the right hand than when clicking alone, in crossed ($t = 6.2$; $p = 0.0001$) and uncrossed ($t = 7.5$; $p = 0.0001$) conditions, consistent with left hand (canonical body) motor extinction when instructed to click with the two hands simultaneously. There was no difference in clicking rate for the *right* hand when used alone vs. when used simultaneously for the crossed ($t = 0.31$; $p = 0.76$) or uncrossed conditions ($t = -1.1$; $p = 0.30$). The rates of the two hands used alone were highly correlated ($r = 0.74$; $p = 0.006$), but were not correlated when used simultaneously ($r = 0.37$; $p = 0.11$). In these cases, patients began using the two hands simultaneously and then prematurely stopped using the left hand, held on either side of space.

#### 3.1.2. Left space motor extinction

No patients showed both types of extinction. Only 7 of 93 patients met criteria for left space motor extinction, by extinguishing clicking of either hand when held in left space when clicking with the two hands simultaneously. For these seven patients, there was no significant difference between rates of the left and right hands when using each alone, although there was again a trend for the left hand to be slower ($t = -2.1$; $p = 0.08$; Table 2). The rates of the two hands used alone were highly correlated ($r = 0.83$; $p = 0.02$), but were not correlated when used together ($r = 0.67$; $p = 0.10$), as the left hand dropped off in this condition. The left hand was significantly reduced when clicking simultaneously in left space than when clicking alone ($t = 2.8$; $p = 0.031$), and the right hand clicking was significantly reduced when clicking simultaneously in left space than when clicking alone ($t = 4.1$; $p = 0.006$), consistent with extinction of either hand used in left space when instructed to click with the two hands simultaneously.

### 3.2. Voxel-based analysis

#### 3.2.1. Left canonical body motor extinction

Extinction of left hand movements, whether clicking in left or right space, was identified in 20 patients (22%). The group of voxels where core infarct was most strongly associated with left canonical body motor extinction across patients by Fisher Exact test was located at Talairach coordinates (x: 30, y: −25, z: 13) in white matter lateral to right thalamus, in the right subcortical temporal cortex (see Fig. 1).

#### 3.2.2. Left space motor extinction

Extinction of movements of either hand held in left space was identified in 7 patients. The group of voxels where tissue dysfunction was most strongly associated with left spatial motor extinction across patients by

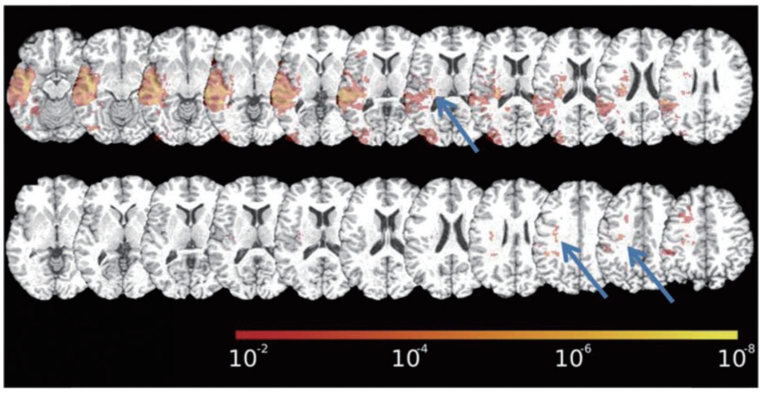

Fig. 1. Statistical map of associations between core infarct and each type of motor extinction using Fisher exact tests. Top panel: map of the association between infarct and presence of left canonical body motor extinction. Bottom panel: map of the association between infarct and presence of left space motor extinction. The color bar shows the corresponding p-values for the association in each voxel. Blue arrows point to the voxel clusters with the highest associations by Fisher exact tests.

Fisher Exact test was located at located in right parietal cortex at Talairach coordinates (x: 30, y:-41, z:30) (see Fig. 1 for voxels where infarct was associated with this deficit).

## 4. Discussion

We found a double dissociation between extinction of movements of the left canonical body (left hand) and extinction of movements by either hand in left space, confirming our hypothesis that these two types of extinction dissociate. In most investigations of motor (as well as visual and tactile) extinction, these two types of extinction in distinct reference frames have been confounded. This observation is important, because it informs theories of the interaction between spatial attention and motor control. Control of bimanual movements in space must depend on two types of dissociable spatial representations or maps, over which attention can be separately disrupted. In one type of spatial representation that guides or maintains movements in space, attention is distributed to the two sides of the body (the two hands) simultaneously. If this representation is disrupted, the patient may have left canonical body motor extinction. In a separate spatial representation that guides or maintains movements in space, attention is distributed to the two sides of space (on each side of the person) simultaneously. If this representation is disrupted, the patient may extinguish movements on the disrupted side of space with either hand held in that space (left spatial motor extinction).

It also seemed that these two dissociable forms of extinction were associated with distinct areas of infarct, although we cannot be too confident about the areas of ischemia associated with each form of extinction, because we cannot be sure of the entire region of ischemic tissue in some of our patients. Not all patients had perfusion-weighted imaging, and both our studies and the investigations of others (e.g. [8,16,17,44,50] have demonstrated that in acute stroke, the deficits depend not only on the area of infarct, but also the surrounding area of hypoperfusion. This finding has been reported most often in patients with subcortical infarcts (e.g. see preceding citations). Left canonical body motor extinction was most strongly associated with infarcts in right subcortical temporal cortex, in white matter lateral to right thalamus. Of those patients who had perfusion-weighted imaging, patients with left canonical body motor extinction often had right temporal cortex hypoperfusion (see illustrative case in Fig. 2). Temporal lesions have previously been associated with allocentric or object-centric neglect, irrespective of the location of the stimulus in space [9,16]. This finding would extend the association between temporal lesions and canonical, location-independent neglect to extinction, often considered a subtle form of neglect. Alternatively, this subcortical lesion site may be critical because of white-matter tracts in this region. For example, thalamocortical pathways have been shown to result in motor neglect [15]. Right motor extinction has previously been described in patients with lesions in the left ventrolateral thalamus, typically due to stereotactic surgery for intractable dyskinesia. Early observations of this phenomenon were characterized by patients who would drop a glass of water held in the contralesional hand, when simultaneously carrying a glass with the ipsilesional hand. This motor extinction typically re-

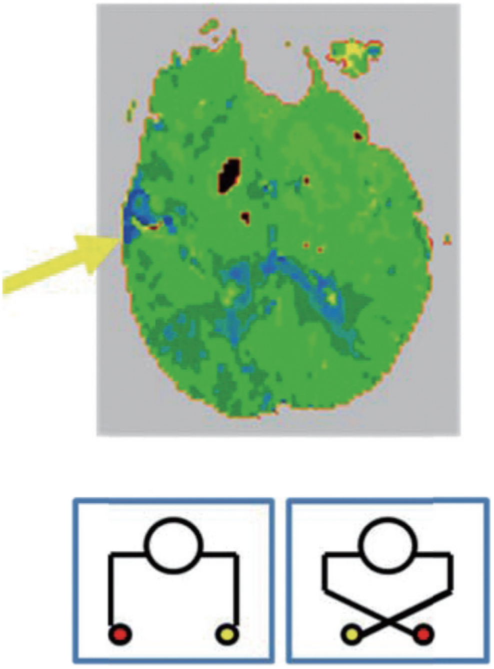

Fig. 2. Top Panel. Perfusion Weighted Image showing 6 sec delay in the time to peak arrival of contrast (which has been demonstrated to be associated with tissue dysfunction; see Shirani et al. [46], also Sobesky et al. [45]) in superior temporal cortex in a patient with left canonical body extinction. Lower Panel. Schematic representation of left canonical body extinction. The yellow hand is extinguished in both the uncrossed (left) and crossed (right) conditions.

solved relatively quickly after surgery [15]. However, previous studies did not attempt to define the reference frame of motor extinction resulting from thalamic lesions. Interestingly, temporoparietal lesions in monkeys produced impairments in using the non-paretic limb contralateral to the lesion to respond to an ipsilesional target, but no impairment in responding with ipsilesional limb to the contralesional target (i.e. motor neglect but no perceptual neglect) [53].

Left space motor extinction was less common, and was associated with infarct in right parietal cortex and frontoparietal white matter. Tissue dysfunction in these regions has previously been associated with neglect of viewer-centered space [7,12,31,33,34,46,50,51]. Parietal lesions have been shown to lead not only to perceptual neglect but also to impairments in directing and maintaining movements in contralateral space [29]. Parietal lesions have also been associated with viewer-centered visual and tactile extinction [16,49,55]. For example, in a detailed analysis of visual extinction both between and within fields, four patients with frontoparietal lesions including dorsal parietal cortex showed extinction in locating but not enumerating figures (star shapes); location but not enumeration performance was influenced by similarity of right-sided stimuli [55]. Performance on left- most targets was worse with right visual field than left visual field simultaneous stimuli. Results indicated that extinction is modulated by task demand; and viewer-centered visual extinction entails a disruption of attention to concurrent, similar targets when features of the targets must be identified. Left space motor extinction may be due to impaired allocation of attention across a viewer-centered spatial representation that directs or maintains movements of the two hands (or other body parts) in space. Extinction has also been ascribed to a hemispheric imbalance, with hyperactivity of attentional neurons in the unaffected hemisphere in the presence of an ipsilesional target (as well as contralesional target) [24–26]. Inhibition of left frontal or parietal cortex with paired transcranial magnetic stimulation (TMS) reduced left tactile extinction patients with right hemisphere stroke, presumably by reducing hyperattention to right space [36]. Similarly, Seyal and colleagues reported that TMS over parietal cortex led to increased sensitivity to ipsilateral cutaneous stimulation, most likely via transient inhibition of ipsilateral parietal cortex and disinhibition of contralateral parietal cortex. Furthermore, inhibitory repetitive TMS to parietal cortex in healthy participants induced visual extinction of targets in contralateral space with simultaneous stimulation in ipsilateral space [38]. Although similar research has not yet been done in the domain of *motor* extinction, the association between left space motor extinction and parietal cortex lesions seems consistent with the previous literature on neural correlates of neglect and extinction in a viewer-centered spatial reference frame.

The second cluster of voxels where lesions were associated with right space motor extinction was in frontoparietal white matter; lesions in this region may have disrupted functional connections between motor regions and spatial representations in parietal cortex that maintain movements in contralateral space. Disconnection of frontal-parietal pathways has been shown to contribute to neglect [11,42,48], and extension of lesions to these subcortical pathways might produce more severe neglect through disturbances of functional connectivity [56]. This study provides additional evidence that parietal cortical areas and corresponding connections with motor pathways in the frontal cortex and basal ganglia might be crucial for a making use of a map of space relative to the side of the "viewer" to guide or maintain movements in space, such that a lesion might cause extinction of movements of the hand held in left space. Although Bartolomeo et al. [4] speculated that extinction might be considered a form of partial callosal interhemispheric disconnection, based on patients with lesions in the corpus callosum who had extinction, our results are more consistent with their notion of (at least left spatial) motor extinction as being due to frontoparietal intrahemispheric disconnection.

We did not find motor extinction associated with areas of the brain devoted to motor planning or motor execution, probably because these areas, when damaged, cause apparent or real left hemiplegia or hemiparesis, which were exclusion criteria.

Future studies will evaluate the entire region of dysfunctional tissue in patients with each form of extinction by obtaining perfusion-weighting imaging in all patients, to fully evaluate the areas of cortical as well as subcortical regions that contribute to each patient's deficits. We also plan to evaluate neural correlates of left canonical body tactile extinction versus left space tactile extinction, by testing tactile extinction in the hands with the hands in crossed and uncrossed conditions. A previous study reported a patient with left canonical body tactile extinction; the patient extinguished tactile stimulation on either left side of the palm as represented in canonical "anatomical position", whether the palm was facing up or down [35]. Others have reported left visual extinction, even when both visual stimuli were presented on the left or right, relative to midline of the viewer [12]. Finally, future studies will need to evaluate various forms of extinction due to left hemisphere stroke. Our previous work indicated that right neglect is as common as left neglect after acute left hemisphere stroke, but right neglect is much more likely to be object- or stimulus-centered than viewer-centered, while left neglect is much more likely to be viewer-centered than object- or stimulus-centered [27].

## 5. Conclusions

There are dissociable types of left motor extinction, distinguished by reference frame– one in which the patient extinguishes movements of the left hand, irrespective of the location of the hand, and one in which the patient extinguishes the either hand held in left space. These two forms of motor extinction appear to be associated with distinct lesion sites in acute stroke, with left space motor extinction due to more dorsal (subcortical parietal or frontoparietal) infarcts and left canonical body extinction due to more ventral (subcortical temporal) infarcts, although the lesion associations require further confirmation.

This study points out the importance of disentangling the different types of motor extinction, because they may have different implications for activities of daily living or rehabilitation. In particular, patients with deficits in an object-centered reference frame of reference, like left canonical body motor extinction, may respond to treatment designed to widen the window of attention to encompass complete objects (e.g. the entire body in this case). Patients with deficits in a viewer-centered reference frame, like left space motor extinction, may respond to treatment focused on shifting attention more to the contralesional side of the viewer (perhaps by reducing hyperattention to the ipsilesional side of space or facilitating disengagement from the ipsilateral space). Interventions that result in a shift of attention include prism adaptation [5,10,28, 39,47], limb activation [40], caloric stimulation [6,19]; neck vibration [21,23]; and trunk rotation and scanning training [57] (see [1,43]; or for review). Whether such interventions results in improvement in left space motor extinction requires investigation.

## Sources of funding

This work was supported by the National Institutes of Health, NIH RO1 NS R01047691 to AH.
